# Dose-dependent evaluation of chronic oleocanthal on metabolic phenotypes and organ toxicity in 5xFAD mice

**DOI:** 10.1016/j.prenap.2025.100357

**Published:** 2025-09-03

**Authors:** Euitaek Yang, Nour F. Al-Ghraiybah, Amer E. Alkhalifa, Lauren N. Woodie, Samuel P. Swinford, Judy King, Michael W. Greene, Amal Kaddoumi

**Affiliations:** a Department of Drug Discovery and Development, Harrison College of Pharmacy, Auburn University; Auburn, AL 36849, USA; b Department of Nutritional Sciences, College of Human Sciences, Auburn University; Auburn, AL 36849, USA; c Department of Basic Sciences, DeBusk College of Osteopathic Medicine, Lincoln Memorial University, Knoxville, TN 37932, USA; d Department of Pharmacology and Toxicology, Medical College of Georgia, Augusta University, Augusta, GA 30912, USA

**Keywords:** Alzheimer’s disease, Oleocanthal, 5xFAD, Amyloid-β, Phenotype changes, Metabolic phenotype, Promethion cages

## Abstract

In Alzheimer’s disease (AD), alterations in the basal metabolic rate (BMR) and energy expenditure, known as metabolic phenotyping, are present early in the disease, which progresses as the disease advances. The Mediterranean diet, including extra-virgin olive oil (EVOO), has been known to reduce AD risk. Oleocanthal (OC) is a major phenolic compound in EVOO. Previous research showed that OC reduced brain amyloid-β, tau hyperphosphorylation, neuroinflammation, and improved blood-brain barrier and memory functions in AD mouse models. In this work, we aimed to investigate the dose-dependent impact of chronic oral OC treatment on modulating metabolic phenotypes affected by AD and its toxicity in 5xFAD mice, an AD mouse model. 5xFAD mice were treated with OC for 3 months, starting at the ages of one (prevention mode, before the pathology hallmarks appear) and 6 months (treatment mode, after the pathology hallmarks appear). Findings demonstrated OC altered metabolic phenotypes in the 5–20 mg/kg dose range in both groups. Furthermore, OC proved not toxic except at 20 mg/kg, where hepatic toxicity is observed. In conclusion, these findings highlight the OC effect in rectifying metabolic phenotypes in AD. However, it limits the dose range in mice to 5 and 10 mg/kg despite exhibiting a favorable response in metabolic parameters due to observed hepatotoxicity with the 20 mg/kg.

## 1. Introduction

Alzheimer’s disease (AD) is a progressive neurodegenerative disorder characterized by cognitive decline and memory loss [1]. Its pathophysiology involves the accumulation of amyloid-β (Aβ) plaques and neurofibrillary tangles (NFTs), leading to synaptic dysfunction and neuronal loss [2]. Hallmarks of AD can be further extended to include oxidative stress, neuroinflammation, blood-brain barrier breakdown, and metabolic dysfunction [3-6]. Metabolic dysfunction includes decreased brain glucose metabolism, suggesting impaired energy utilization [6,7]. While familial AD is linked to specific genetic mutations, sporadic AD, which constitutes most AD cases, is influenced by a combination of genetic, environmental, and lifestyle factors, with the apolipoprotein E (APOE) ε4 allele being a significant genetic risk factor [8]. Patients with AD often exhibit various neuropsychiatric symptoms, including depression, anxiety, and sleep disturbances, which significantly affect their quality of life [9]. The complexity of AD’s pathogenesis underscores the challenge of developing effective treatments and necessitates ongoing research into its multifaceted nature.

The interplay between lifestyle modifications and the prevention of AD is a subject of increasing scientific interest. Research has demonstrated that lifestyle choices, encompassing diet, physical activity, and cognitive engagement, can significantly influence the risk and progression of AD [10,11]. The Mediterranean diet, characterized by high consumption of fruits, vegetables, whole grains, and particularly extra-virgin olive oil (EVOO) alongside moderate fish intake, has been linked to a reduced risk of cognitive decline [12-14]. This diet is rich in antioxidants and anti-inflammatory agents, which are thought to offer neuroprotective effects against the development of AD [15-17].

EVOO is a source of monounsaturated fats and is abundant in phenolic compounds, including oleocanthal (OC) [18]. OC, a naturally occurring phenolic secoiridoid, is recognized for its anti-inflammatory and antioxidant properties, which mirrors the effects of ibuprofen, suggesting potential therapeutic roles in AD [19-23]. Studies from our laboratory and others demonstrated OC interferes with the aggregation of Aβ proteins, an essential process in the pathogenesis of AD, and modulates pathways involved in inflammation and oxidative stress [21,22,24,25]. These findings suggest OC could be used as a therapeutic molecule or as a dietary supplement.

While typical dietary levels of OC, as found in EVOO, are generally deemed safe, the effects of higher doses for potential nutraceutical use, especially in AD, remain underexplored [23,26,27]. Some studies suggest that elevated levels of OC could exert cytotoxic effects on specific cell types, including neuronal cells, highlighting the importance of understanding the dose-dependent impacts of this compound in vivo [28]. The safety of OC was evaluated by Siddique et al., 2020, in which a dose-dependent acute toxicity profile was assessed in wild-type mice. In this study, Swiss albino mice were treated with a single dose of oral OC (10, 250, or 500 mg/kg), followed by biochemical, hematological, and histological examinations [23]. At a foundational level, a single 10 mg/kg oral dosage of OC was deemed safe for Swiss albino mice, exhibiting no discernible toxicity in the brain, lung, liver, spleen, pancreas, and small intestine. Nonetheless, histopathological analyses showed that elevated single dosage at 250 and 500 mg/kg may provoke modest adverse impacts, notably affecting this species’ cardiac and renal systems [23]. However, it remains to elucidate the safety and toxicity profile of chronic OC using in vivo models, which makes it imperative to evaluate its safety and efficacy comprehensively.

The metabolic phenotype in AD is a critical aspect that includes a range of metabolic parameters reflecting individual metabolic functions [29,30]. Metabolic parameters encompass energy expenditure, which is crucial for understanding calorie consumption and the utilization rates of carbohydrates and fats. Energy expenditure in AD patients is measured through various variables, including oxygen consumption, carbon dioxide production, and the respiratory quotient (RQ). RQ, the ratio of carbon dioxide volume produced to oxygen volume consumed at the cellular level, along with the respiratory exchange ratio (RER) which assesses the ratio in the whole body, is a crucial indicator of metabolic efficiency [31].

In AD, there are alterations in the basal metabolic rate (BMR), the rate of energy expenditure while at rest, which is calculated to estimate daily caloric needs, factoring in physical activity levels [32,33]. The metabolic profile of an AD patient also includes changes in water and food consumption, sleep duration, movement distance, and body weight, all of which are essential in understanding the overall metabolic impact of the disease [34,35]. These changes are often accompanied by sleep disturbances and reduced meal consumption, leading to malnutrition, dehydration, disrupted homeostasis, compromised immune function, and further cognitive decline [35,36]. Such sleep disturbances are symptoms and exacerbate the disease’s progression by disrupting circadian rhythms and affecting the glymphatic clearance of Aβ [37,38].

Previously, we have evaluated the effects of AD pathology in 5xFAD mice on altering metabolic parameters compared to cognitively normal mice [35]. In addition, we assessed the impact of three months of treatment of 10 mg/kg oral OC in mice as a function of age and pathology [35]. Our findings demonstrated a significant difference in metabolic parameters between wild-type and 5xFAD mice. Moreover, OC treatment reduced anxiety-like behavior, namely, activity rate, movement, and sleep disturbances in 5xFAD mice, reaching levels similar to age-matched wild-type mice. Besides, OC treatment reduced age-dependent behavioral changes in both wild-type and 5xFAD mice [35]. In the current work, we aimed to investigate the dose-dependent effect of OC on metabolic phenotype changes and its toxicity on various organs. We employed the 5xFAD mouse model and conducted dose-dependent experiments over two groups of mice who received daily OC for 3 months. Our findings revealed more pronounced changes in metabolic parameters among older mice with advanced AD pathology than their younger counterparts that were modulated by OC in the evaluated dose range. Additionally, using histopathology, OC administered at 20 mg/kg demonstrated significant hepatic toxicity.

## 2. Materials and methods

### 2.1. Animals

Male wild-type (WT) C57BL/6 J (Strain#: 000664) and 5xFAD (strain#: 034848-Jax) mice were obtained from The Jackson Laboratory (Bar Harbor, ME, USA). The genetic background of this 5xFAD strain is C57BL/6 J. All animal experiments and procedures followed the guidelines of the Institutional Animal Care and Use Committee of Auburn University (Protocol number 2017–3179) and adhered to the principles outlined by the National Institutes of Health for laboratory animal care and followed the ARRIVE guidelines. The 5xFAD mouse model expresses the human amyloid precursor protein (APP) with the mutations APP KM670/671NL (Swedish), APP I716V (Florida), APP V717I (London), PSEN1 M146L, and PSEN1 L286V, resulting in early and aggressive accumulation of Aβ at the age of two to three months with subsequent spatial learning deficits as the disease progresses by the ages three to six months that worsen with age. 5xFAD mice start developing microvesicular inflammation by four months, becoming more prevalent at nine to twelve months [39-41]. Therefore, for this work, we used mice at one and six months as representative ages to evaluate OC for prevention vs treatment modes, respectively.

**Fig. 1.**
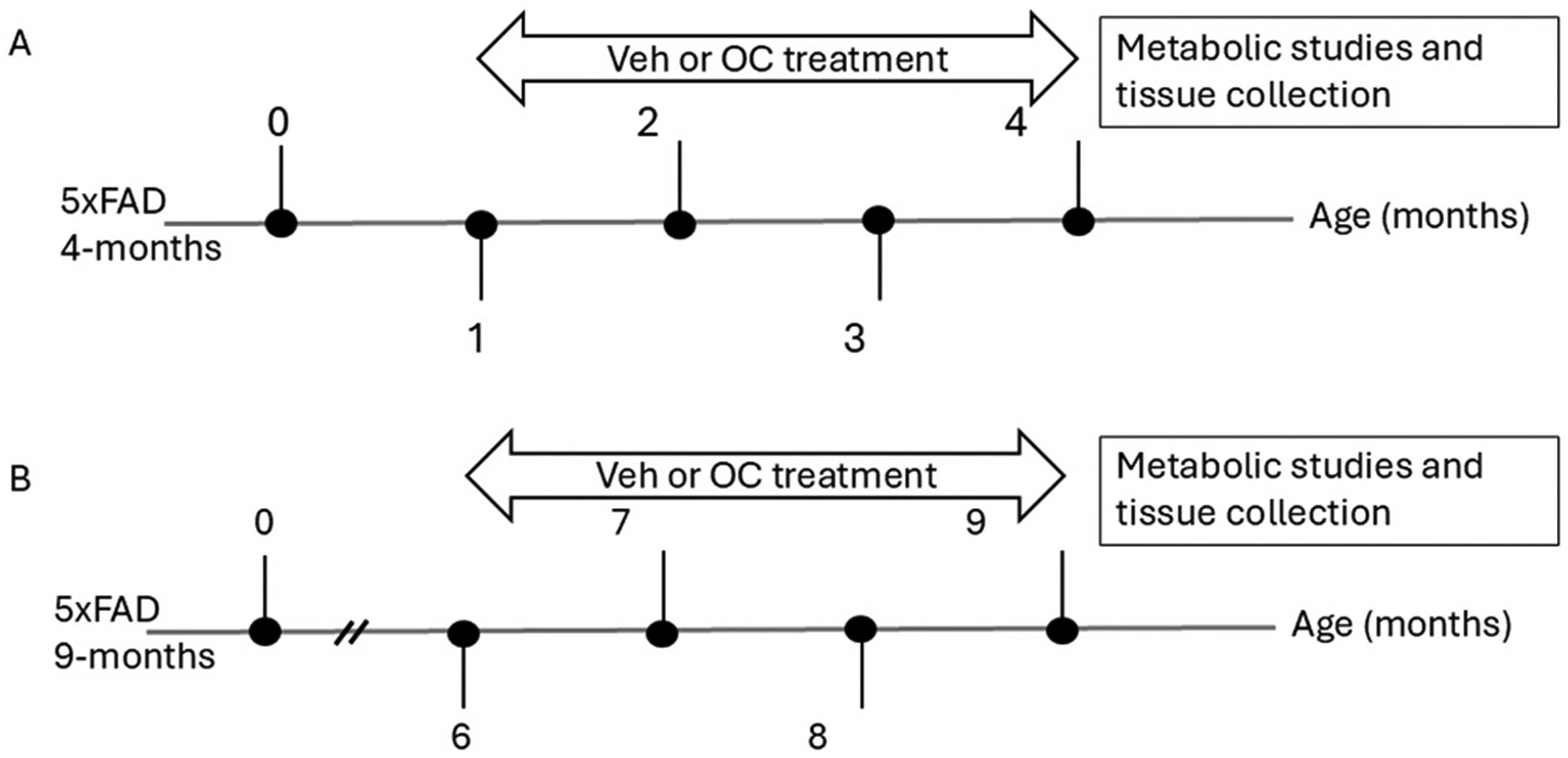
A schematic presentation of the experimental design. This study used two age groups (A) four-month-old male mice with treatment started at the age of one month, and B) nine-month-old male mice with treatment started at the age of six months, 5xFAD mice. Mice in each group were treated with saline as the vehicle (Veh) or OC for three months, followed by metabolic studies and tissue collection. Four months and nine months WT mice were also used in the study for comparison.

OC with purity > 99 % was obtained from Oleolive LLC (Shreveport, LA, USA). Mice treatment started at one and six months of age for three months, ending the treatment at four and nine months old, respectively. 5xFAD mice received daily treatment of OC via oral gavage of 0 (saline as a vehicle), 0.5, 1, 2.5, 5, 10, and 20 mg/kg for three months (n = 10 mice per dose per group). Age-matched WT mice (n = 10 mice), as the control group, received saline as the vehicle. All mice received a volume of 100 μl/30 g (or 3.3 mL per kg) body weight of the solution treatment within 15 sec as the duration of administration. Mice were housed in plastic cages under a 12-hour light/dark cycle, 22°C temperature, 35 % relative humidity, and ad libitum access to food and water. At four and nine months, the mice were transferred to metabolic cages to assess metabolic and behavioral phenotypes, followed by tissue collection. Study design and time frames are represented in Fig. 1.

### 2.2. Measurement of metabolic parameters

After three months of OC treatment, changes in metabolic phenotype parameters were monitored using the Promethion Metabolic Mouse Cages (Sable Systems, Las Vegas, NV). For data collection, the mice were transferred to metabolic cages, followed by data collection as we previously reported [35]. Each cage housed one mouse for 36 h; the first 12 h were for acclimation, followed by consecutive 12 h of daytime and 12 h of nighttime data collection. Metabolic parameters were assessed over a 12 h:12 h light:dark cycle with zeitgeber time (ZT) 0 (representing lights on) and ZT12 (representing lights off). Animals’ activity was monitored via Promethion XYZ Beambreak Activity Monitor. Phenotypic parameters were registered through Promethion precision MM-1 Load Cell sensors. Table 1 summarizes the collected metabolic parameters and their definitions. Respirometry data were analyzed using the Promethion GA-3 gas analyzer to provide comprehensive respirometry data. All metabolic phenotyping data were analyzed using ExpeData software (version 1.8.2; Sable Systems) with Universal Macro Collection (version 10.1.3; Sable Systems).

### 2.3. Tissue collection

Following the metabolic parameters collection, mice were anesthetized using intraperitoneal injections of xylazine and ketamine (20 and 125 mg/kg, respectively), followed by decapitation. Mouse brains were extracted, and the two hemispheres of each brain were separated for immunostaining and biochemical analysis, and stored at −80°C. Other tissues collected are the kidney, spleen, liver, small intestine, and large intestine. Tissues were fixed as whole organs using 70 % ethanol. After fixation, tissues were dehydrated using an ethanol gradient of 70–100 %, followed by xylene treatment, infiltrated with wax, and embedded in paraffin. At the staining time, tissues were deparaffinized and hydrated using xylene, followed by a descending ethanol gradient, ending with a water wash. Tissues were stained with hematoxylin (3 min), followed by mild acid differentiation, bluing, and eosin staining (45 s). Tissues were then dehydrated by ascending ethanol washes ending with xylene and slide mounting. Slides were visualized using an Olympus BX41 light microscope with CellSens imaging software (Olympus USA). Magnification for the kidney, liver, and intestine was 200X, and for the spleen was 100X. For the histopathological analysis, we analyzed 2 sections per organ per mouse for n = 3 mice per dose. A complete field per section was imaged and analyzed.

### 2.4. ELISA and Aβ staining

Brain homogenates were used to evaluate soluble Aβ levels. Commercially available ELISA kits for Aβ_40_ and Aβ_42_ were used according to the manufacturer’s instructions (R&D Systems, Minneapolis, MN). All samples were run in duplicate. For Aβ plaques staining, brain sections of 14 μm thickness were fixed with 4 % formaldehyde, followed by thioflavin-S (ThioS) staining to visualize Aβ plaques as reported previously [25].

### 2.5. Statistical analysis

Data analysis of metabolic and behavioral parameters was performed using IBM SPSS Statistics 25 (Armonk, New York). Data were checked for normality and sample homogeneity and analyzed using ANOVA with Dunnett’s post-hoc test. A series of one-way ANOVAs with a 1 × 8 between-subjects design, where the independent variable was the treatment group with 8 levels (WT, 5xFAD, and treatment groups), and each ANOVA examined one dependent variable at a time (e.g., VO2, VCO2, etc.). Dunnett’s post-hoc tests were run to compare treatment groups with WT mice or vehicle-treated 5xFAD mice. Data were graphed using GraphPad Prism (San Diego, CA). Statistical significance was determined at p < 0.05; all data are presented as Mean ± SEM for n = 10 mice per group.

## 3. Results

### 3.1. Effect of oleocanthal on metabolic parameters in 5xFAD mouse model: prevention approach

**Fig. 2.**
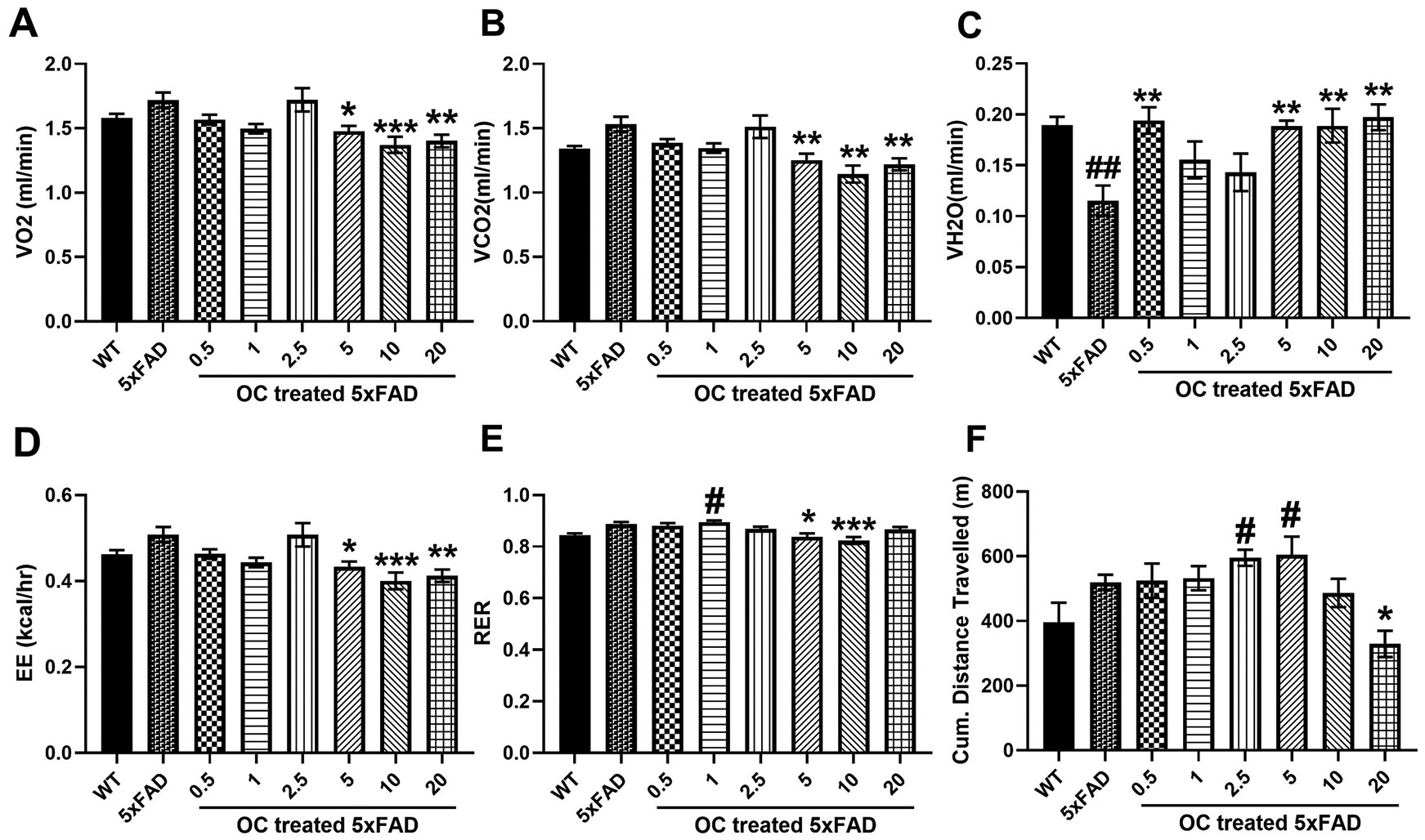
The effect of OC treatment on the metabolic parameters in four-month-old mice at nighttime. (A) VO_2_ (mL/min), (B) VCO_2_ (mL/min), (C) VH_2_O (mL/min), (D) EE (kcal/h), (E) RER, and (F) Cumulative (Cum.) distance traveled in WT and 5xFAD mice. The statistical significance compared to WT is coded with # or * if significant to non-treated 5xFAD mice. Data are presented as mean ± SEM for n = 10 per group with *p < 0.05, **p < 0.01, and ***p < 0.001 compared to vehicle-treated 5xFAD mice, and #p < 0.05 and ##p < 0.01 compared to vehicle-treated WT mice.

**Fig. 3.**
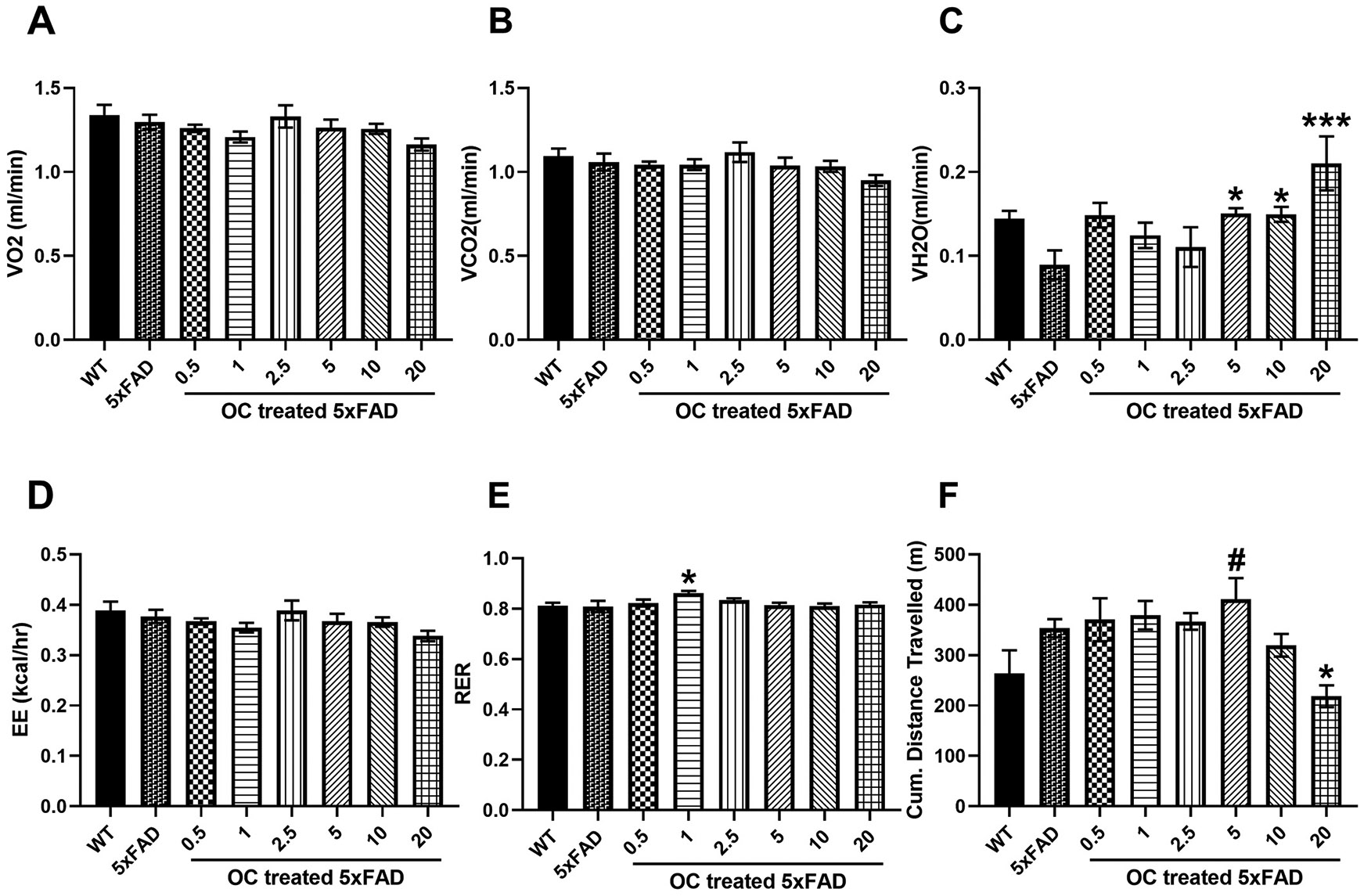
The effect of OC treatment on the daytime metabolic parameters in four-month-old mice. (A) VO_2_ (mL/min), (B) VCO_2_ (mL/min), (C) VH_2_O (mL/min), (D) EE (kcal/h), (E) RER, and (F) Cumulative (Cum.) distance traveled in WT and 5xFAD mice. Data are presented as mean ± SEM for n = 10 per group with *p < 0.05, and ***p < 0.001 compared to vehicle-treated 5xFAD mice, and #p < 0.05 compared to vehicle-treated WT mice.

Figs. 2 and 3 demonstrate the effect of 3 months of OC treatment starting at the age of one month until four months of age in 5xFAD mice’s metabolic phenotypes at night (between 6 pm to 6 am) - and day-(between 6 am to 6 pm) times, respectively. Phenotype measurements were separated into the day- and night- times. The one-way ANOVA parameters (F and p values, and degrees of freedom) of the 8 groups (WT, 5xFAD, and treatment groups) for each measured variable are described in Supplementary Table 1. Mice are nocturnal animals and are more active at night. At nighttime, when the mice are active, no significant difference was observed between WT and vehicle-treated 5xFAD mice in VO_2_ and VCO_2_ parameters. However, a trend of increase was detected in 5xFAD in both parameters (Figs. 2A and 2B). 5xFAD mice treatment with OC significantly reduced VO_2_ by 13.9–20.3 % and VCO_2_ by 18.3–25.5 % in the dose range between 5 and 20 mg/kg when compared to vehicle-treated 5xFAD mice but not with WT mice (Figs. 2A, 2B). VH_2_O in 5xFAD mice was 36.8 % less than in WT mice (Fig. 2C), suggesting a lower dehydration in young 5xFAD mice compared to WT mice, which could point towards the pathological effect in the reduced VH_2_O. The effect of OC was evident in 5xFAD mice treated with a 0.5 mg/kg dose and higher doses, which restored VH_2_O levels to values similar to those in WT mice. OC-treated 5xFAD mice had significantly 58 – 66 % higher VH_2_O than vehicle-treated 5xFAD mice (Fig. 2C). Changes in VO_2_, VCO_2_, and VH_2_O were reflected in EE, where 5xFAD mice treated with 5–20 mg/kg OC had 15.7–21.6 % less EE than vehicle-treated 5xFAD mice, values that are comparable to WT mice (Fig. 2D). No significant difference was observed between WT and vehicle-treated 5xFAD mice for the RER. However, comparing OC treatment to vehicle-treated 5xFAD mice, a small but significant effect was observed, where RER was reduced by 6–8 % in 5 and 10 mg/kg OC (Fig. 2E). Finally, the cumulative distance traveled by mice at nighttime showed a trend of increase in 5xFAD mice and was restored to WT mice levels only with 20 mg/kg OC (Fig. 2F).

During the daytime, there was no significant difference in VO_2_ and VCO_2_ between WT and 5xFAD mice. In addition, OC treatment didn’t change both parameters when compared to vehicle-treated 5xFAD mice (Fig. 3A, B). Whereas VH_2_O increased in 5 and 10 mg treated mice by 66 % and in the 20 mg/kg OC treated mice by 133 % compared to vehicle-treated 5xFAD mice, reaching values similar to WT mice, respectively (Fig. 3C). EE parameter was not altered by early pathology in 5xFAD mice compared to WT mice or with OC treatment (Fig. 3D). For the RER parameter, no meaningful change was observed by OC treatment (Fig. 3E). Although there is a trend of higher cumulative distance traveled by vehicle or OC-treated 5xFAD mice during the daytime compared to WT mice, only 20 mg/kg OC reduced the cumulative distance traveled by ~38 %, reaching levels comparable to WT mice (Fig. 3F).

We also monitored food and water intake, as well as body weight. There was no significant difference in the body weight (Supplementary Figure 1A), and nighttime and daytime food intake (Supplementary Figure 2 A & C) between WT, 5xFAD, and OC-treated mice. Nighttime water intake did not change after treatment (Supplementary Figure 2B). However, daytime water intake in the 10 mg/kg treated 5xFAD mice was 15 % higher than vehicle-treated and other OC-treated 5xFAD mice (Supplementary Figure 2B and D).

### 3.2. Effect of oleocanthal on metabolic parameters in 5xFAD mouse model: Treatment approach

**Fig. 4.**
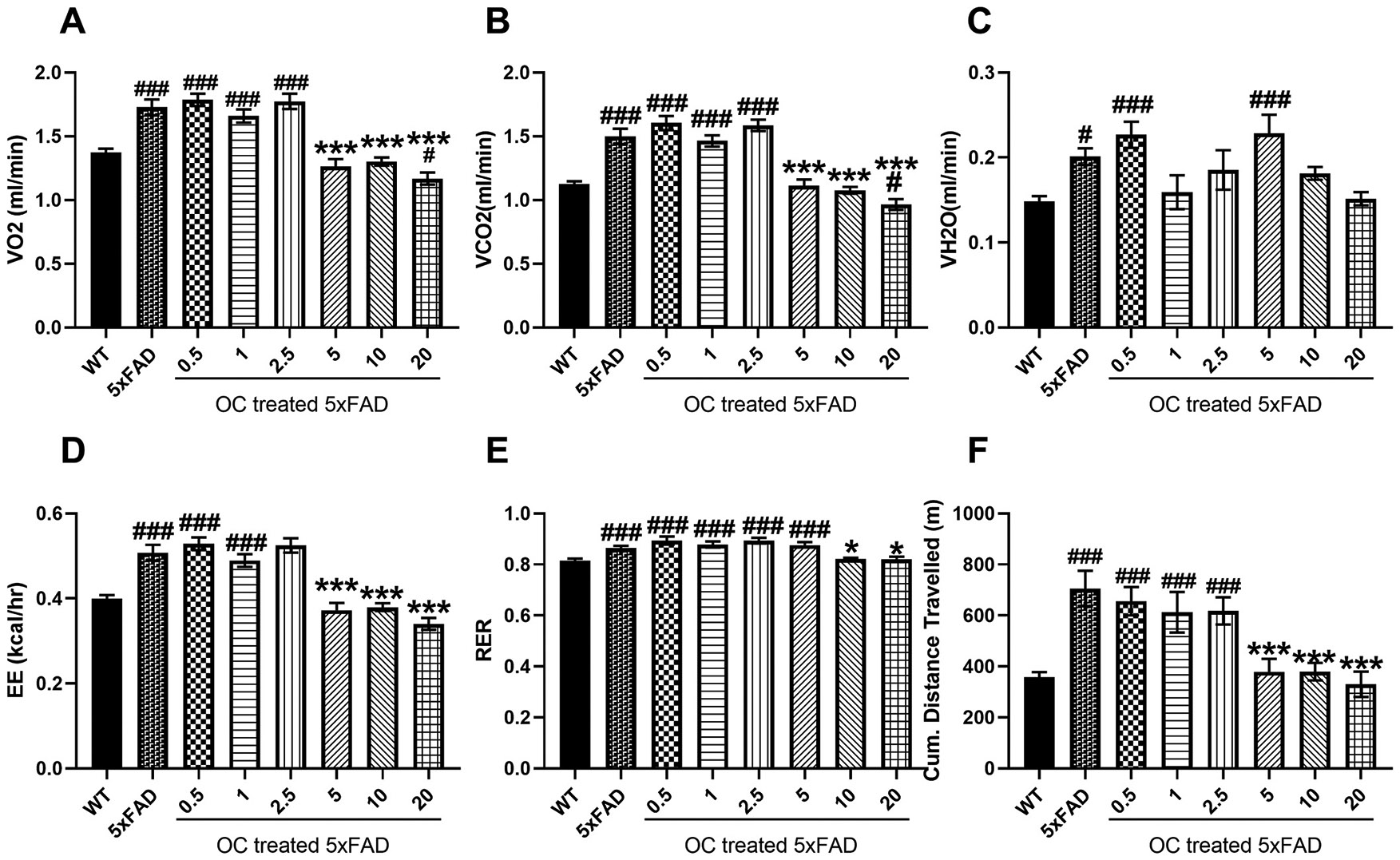
The effect of OC treatment on the metabolic parameters in nine-month-old mice at nighttime. (A) VO_2_ (mL/min), (B) VCO_2_ (mL/min), (C) VH_2_O (mL/min), (D) EE (kcal/h), (E) RER, and (F) Cumulative (Cum.) distance traveled in WT and 5xFAD mice. Data are presented as mean ± SEM for n = 10 per group with *p < 0.05, and ***p < 0.001 compared to vehicle-treated 5xFAD mice, and #p < 0.05 and ###p < 0.001 compared to vehicle-treated WT mice.

The effect of OC treatment was more evident in the treatment approach in 5xFAD mice with advanced AD pathology at the age of nine months following three months of treatment. At nighttime (Fig. 4), the 5xFAD mice have significantly higher VO_2_ (Fig. 4A) and VCO_2_ (Fig. 4B) compared to WT mice. 5xFAD mice treatment with 0.5, 1, and 2.5 mg/kg OC didn’t alter either parameter compared to vehicle-treated 5xFAD; however, they were markedly reduced with 5 and 10 mg/kg OC by ~ 30 %, reaching levels similar to the WT mice. Meanwhile, 20 mg/kg OC significantly reduced VO_2_ and VCO_2_ compared to WT and 5xFAD mice (Fig. 4A and B). The parameter VH_2_O was significantly higher in 5xFAD compared to WT mice by 35 % (Fig. 4C). Although 5xFAD mice treatment with OC in the dose range 0.5–20 didn’t provide a significant effect compared to the vehicle-treated 5xFAD, OC treatment at 1, 2.5, 10, and 20 demonstrated a reduction trend to levels close to WT mice. The one-way ANOVA parameters (F and p values, and effect size) of the 8 groups (WT, 5xFAD, and treatment groups) for each measured variable are described in Supplementary Table 1.

The observed changes in VO_2_, VCO_2,_ and VH_2_O were reflected in the EE parameter. While there was no significant difference between the vehicle-treated 5xFAD and 0.5–2.5 mg/kg-treated OC 5xFAD mice, the EE parameter was significantly higher than WT mice by 22–32 %. OC was effective at the higher doses where, in the range of 5–20 mg/kg, OC significantly reduced EE to comparable levels to WT mice (Fig. 4D).

The RER parameter demonstrated a significantly higher value in vehicle- and 1–5 mg/kg OC-treated mice than in WT mice. Meanwhile, 5xFAD mice treatment with higher doses of OC, 10 and 20 mg/kg, significantly reduced RER to levels comparable to those of WT mice (Fig. 4E). Further, the cumulative distance traveled by mice was > 50 % higher in the vehicle- and 0.5–2.5 mg/kg OC-treated 5xFAD mice than WT mice. However, treatment with OC in the 5–20 mg/kg range significantly reduced the cumulative distance traveled, reaching levels comparable to those of WT mice (Fig. 4F).

**Fig. 5.**
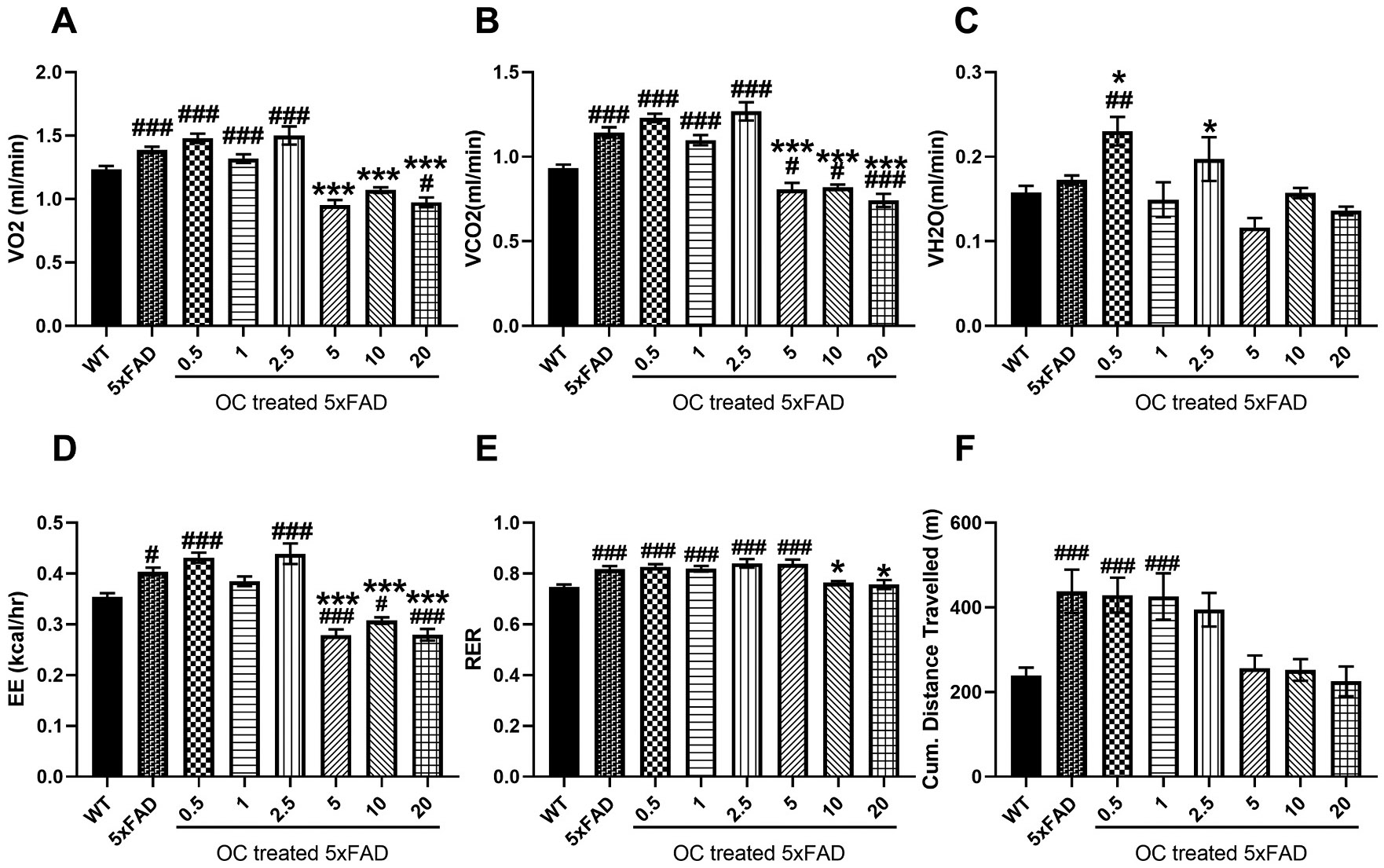
The effect of OC treatment on the metabolic parameters in nine-month-old mice at daytime (A) VO_2_ (mL/min), (B) VCO_2_ (mL/min), (C) VH_2_O (mL/min), (D) EE (kcal/h), (E) RER, and (F) Cumulative (Cum.) distance traveled in WT and 5xFAD mice. Data are presented as mean ± SEM for n = 10 per group with *p < 0.05, and ***p < 0.001 compared to vehicle-treated 5xFAD mice, and #p < 0.05, ##p < 0.01, and ###p < 0.001 compared to vehicle-treated WT mice.

Phenotypic changes during the daytime are presented in Fig. 5, which demonstrates similar changes to those observed during the nighttime. Compared to WT mice, VO_2_ (Fig. 5A) and VCO_2_ (Fig. 5B) were significantly higher in 5xFAD mice by 12 % and 23 %, respectively. 5xFAD mice treated with OC in the dose range between 0.5 and 2.5 mg/kg didn’t alter either parameter’s levels; however, OC doses in the 5–20 mg/kg range significantly reduced both parameters to levels lower than WT mice by approximately 25 %. The VH_2_O parameter was not significantly different between WT and vehicle and OC-treated 5xFAD mice (Fig. 5C) except with 0.5 and 2.5 mg/kg OC, which were significantly higher than 5xFAD and WT mice. Furthermore, the parameters EE, RER, and cumulative distance traveled demonstrated higher levels in 5xFAD mice compared to WT mice, which were not affected by OC treatment in the low dose range (0.5–2.5 mg/kg for EE and 0.5–1 mg/kg cumulative distance traveled, Fig. 5D and F; and 0.5–5 mg/kg for RER, (Fig. 5E). However, at higher doses of OC, EE was significantly reduced to levels lower than 5xFAD and WT (Fig. 5D), RER was reduced significantly by ~10 % compared to vehicle-treated 5xFAD mice, reaching levels comparable to WT mice (Fig. 5E), and cumulative traveled distance was reduced to levels comparable to WT mice without showing significant effect compared to vehicle-treated 5xFAD mice (Fig. 5F).

With regard to the effect of OC treatment on mice’s body weights, vehicle-treated 5xFAD mice and 5 and 20 mg/kg OC-treated 5xFAD mice had 19 % and 15 % less body weight compared to WT mice, respectively. With the other doses, OC increased the mice’s body weight by about 20 % compared to vehicle-treated 5xFAD mice (Supplementary Figure 1B).

In addition, OC treatment didn’t show a significant difference in food intake between the groups when monitored at day and night times (Supplementary Figure 3 A and 3 C). Similarly, OC treatment did not significantly affect water intake during the daytime or nighttime (Supplementary Figure 3B and 3D).

### 3.3. Effect of oleocanthal on sleep pattern in 5xFAD mouse model

**Fig. 6.**
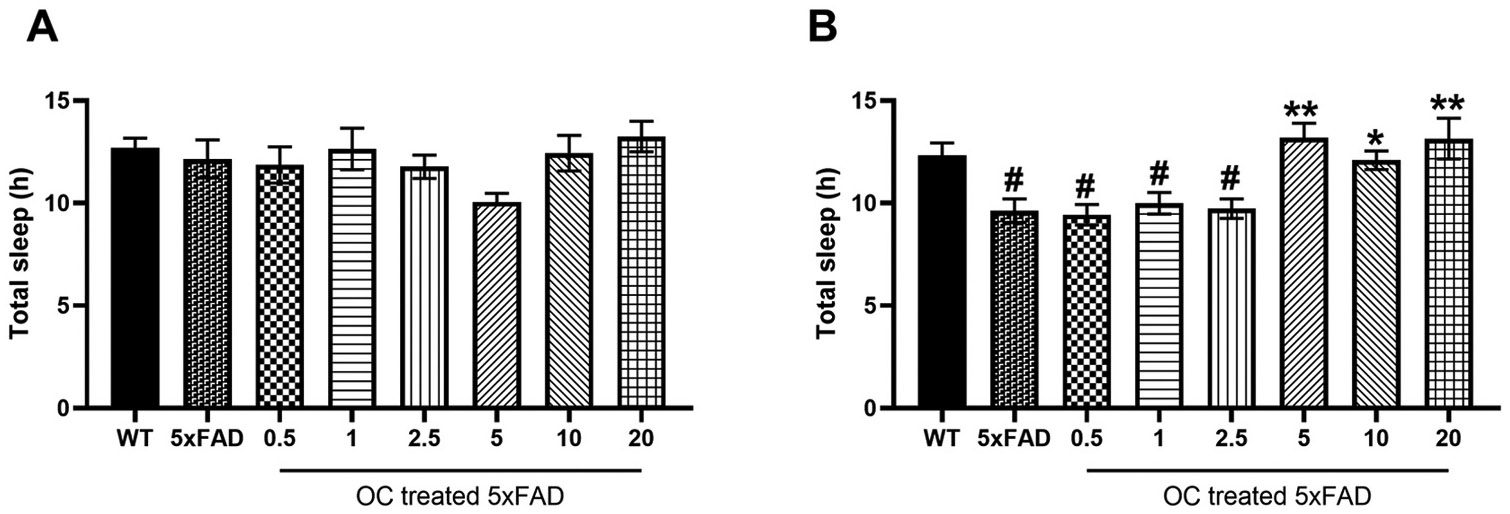
The effect of OC treatment on total sleep time (h) in (A) four-month-old mice, and (B) nine-month-old mice. Data are presented as mean ± SEM for n = 10 per group with *p < 0.05 and **p < 0.01 compared to vehicle-treated 5xFAD mice, and #p < 0.05 compared to vehicle-treated WT mice.

We evaluated the effect of OC on sleep patterns in both early and advanced pathology. In early AD pathology, no significant changes were observed in total sleep between WT, 5xFAD, and OC treatments (Fig. 6A). In the nine-month-old 5xFAD mice with the advanced pathology group, three months of treatment with OC significantly increased total sleep hours in the dose range of 5–20 mg/kg (Fig. 6B).

### 3.4. Effect of oleocanthal on brain Aβ Load in 5xFAD mouse model

**Fig. 7.**
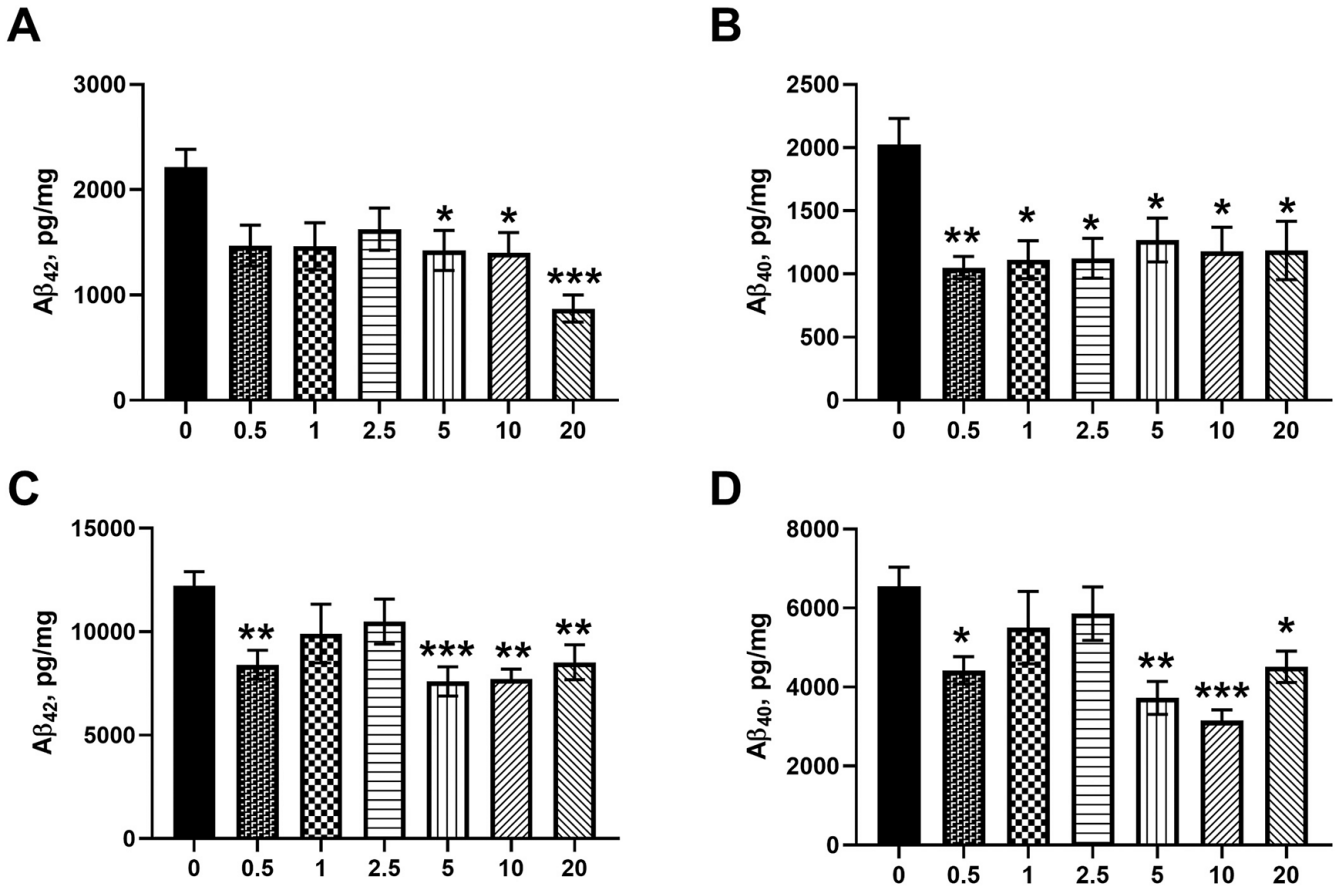
The effect of OC treatment on 5xFAD mouse brain levels. (A) Aβ_42_ and (B) Aβ_40_ in four-month-old 5xFAD mice. (C) Aβ_42_ and (D) Aβ_40_ in nine-month-old 5xFAD mice as determined by ELISA. Data are presented as mean ± SEM for n = 10 per group with *p < 0.05, **p < 0.01, and ***p < 0.001 compared to vehicle-treated 5xFAD mice.

**Fig. 8.**
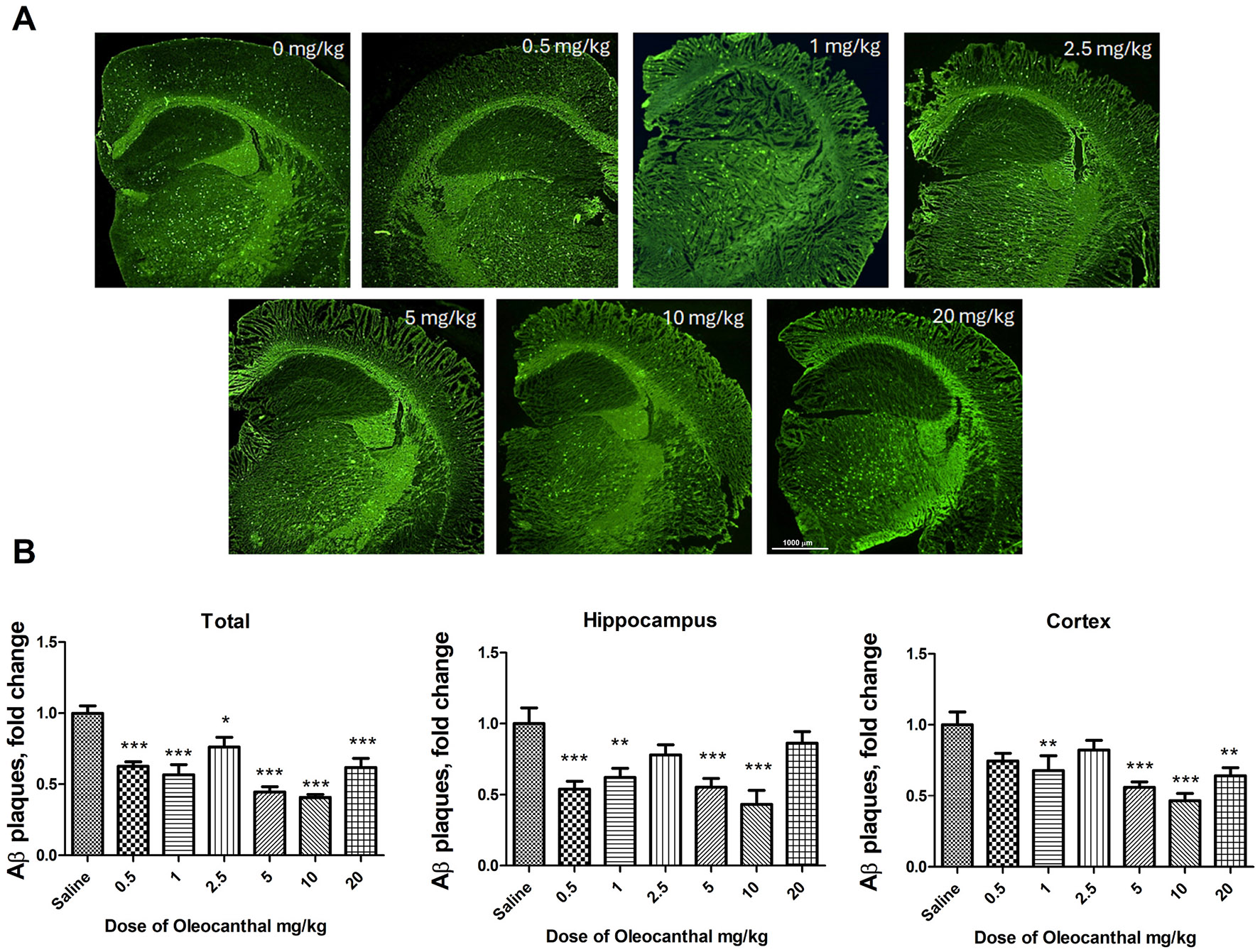
Oleocanthal dose-dependent effect on (A) Aβ deposit (plaques) in the brains of nine-months 5xFAD mice as determined by ThioS staining, and (B) quantification of the number of total Aβ plaques, and in the hippocampus and cortex regions, presented as fold change compared to vehicle-treated 5xFAD mice. Data are presented as mean ± SEM for n = 10 per group with *p < 0.05, **p < 0.01, and ***p < 0.001 compared to vehicle-treated 5xFAD mice.

Brain Aβ levels were evaluated in both mouse groups. ELISA and fluorescence imaging data of brain Aβ levels are shown in Figs. 7 and 8. Fig. 7 shows the soluble Aβ40 and Aβ42 levels in brain homogenates measured by ELISA in four- and nine-month-old mice treated with OC. In the prevention group represented by the four-month-old 5xFAD mice, a trend of reduced levels of Aβ_42_ in the OC dose range 0.5–2.5 mg/kg was observed when compared to vehicle-treated 5xFAD mice, and the OC effect reached a significant reduction in the dose range 5–20 mg/kg where OC reduced Aβ_42_ by 30–60 % compared to vehicle-treated 5xFAD mice (Fig. 7A). Whereas all doses of OC (0.5–20 mg/kg) significantly reduced Aβ_40_ levels by 40–50 % (Fig. 7B). In the treatment group represented by the 9-month-old 5xFAD mice, brain Aβ_42_ and Aβ_40_ were reduced by at least 30 % in 0.5 and 5–20 mg/kg OC treatments (Fig. 7C, D). While additional studies are necessary to explain this result, in the treatment mode group, OC at 0.5 mg/kg was able to reduce brain Aβ levels, but not with 1 and 2.5 mg/kg, which were ineffective in Aβ reduction. Overall results suggest that higher doses (5–20 mg/kg) could be required for advanced pathology when compared to the prevention group with less pathology. Moreover, to visualize the reduction in Aβ plaques, Fig. 8A shows ThioS-stained brain tissues at different OC doses with their quantification in Fig. 8B. No plaques were observed in the brains of mice of the prevention group (4-month-old 5xFAD mice, data not shown). In the nine-month-old mice with advanced pathology, OC treatment significantly reduced total brain Aβ plaques in the 0.5–20 mg/kg dose range. We also evaluated the dose-dependent effect of OC treatment in mice hippocampi and cortexes, where OC significantly reduced Aβ plaques in mice brains compared to vehicle-treated 5xFAD mice.

### 3.5. Effect of oleocanthal on organ toxicity in 5xFAD mouse model

**Fig. 9.**
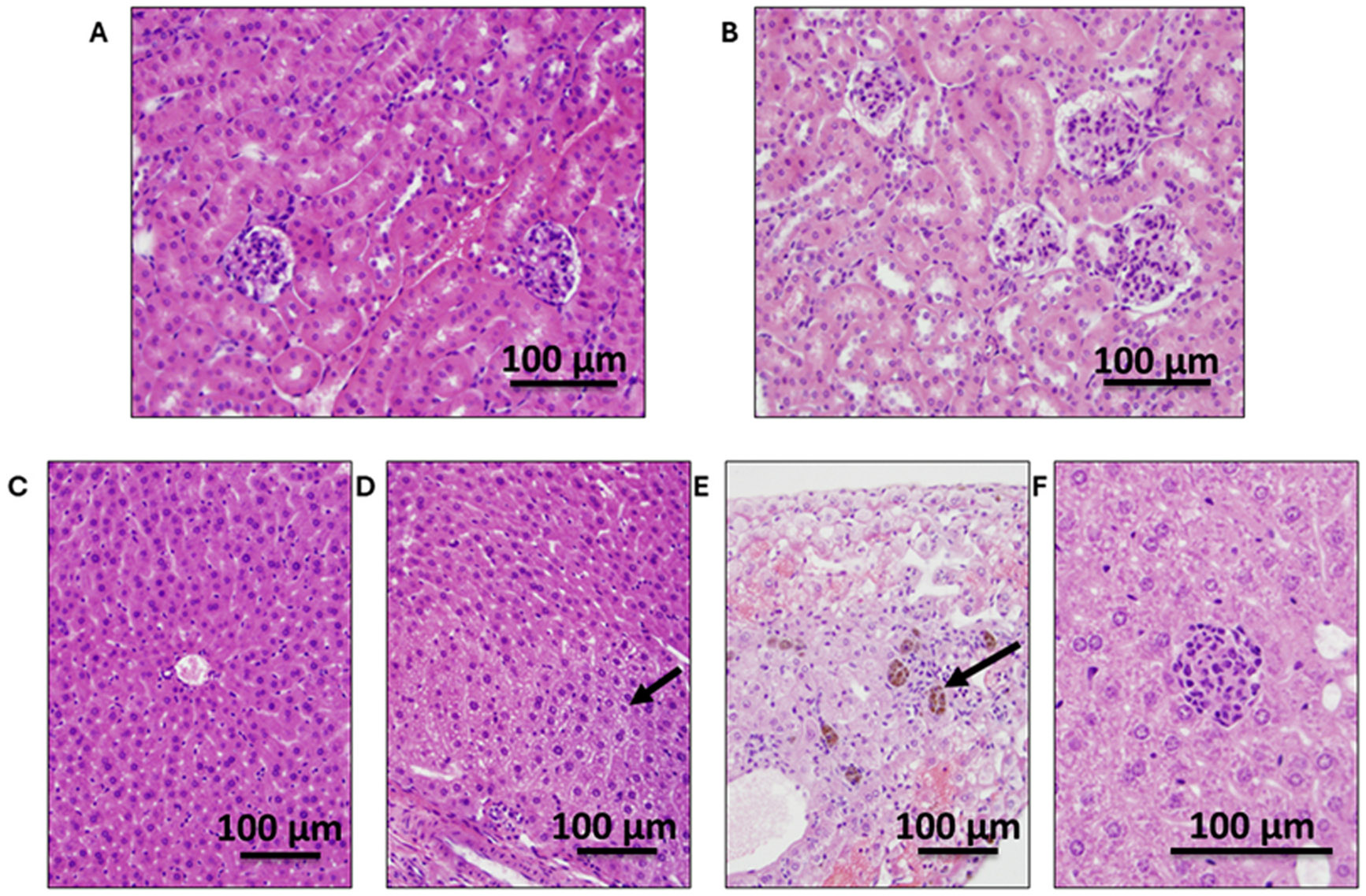
Representative sections for the effect of OC on organ toxicity in four-month-old 5xFAD mice. (A) Kidney section from vehicle-treated 5xFAD mouse (20x objective; 200x), (B) kidney section from 5xFAD mouse receiving 20 mg/kg OC (20x objective; 200x), (C) liver section of vehicle-treated WT mouse (20x objective; 200x), (D) liver section from vehicle-treated 5xFAD mouse showing focal microvesicular steatosis (arrow) (20x objective; 200x), (E) liver section from 5xFAD mouse receiving 20 mg/kg OC showing focal hemosiderin-laden cells (arrow) (20x objective; 200x), and (F) liver section from 5xFAD mouse receiving 20 mg/kg OC showing focal chronic inflammation (40x objective; 400x).

**Fig. 10.**
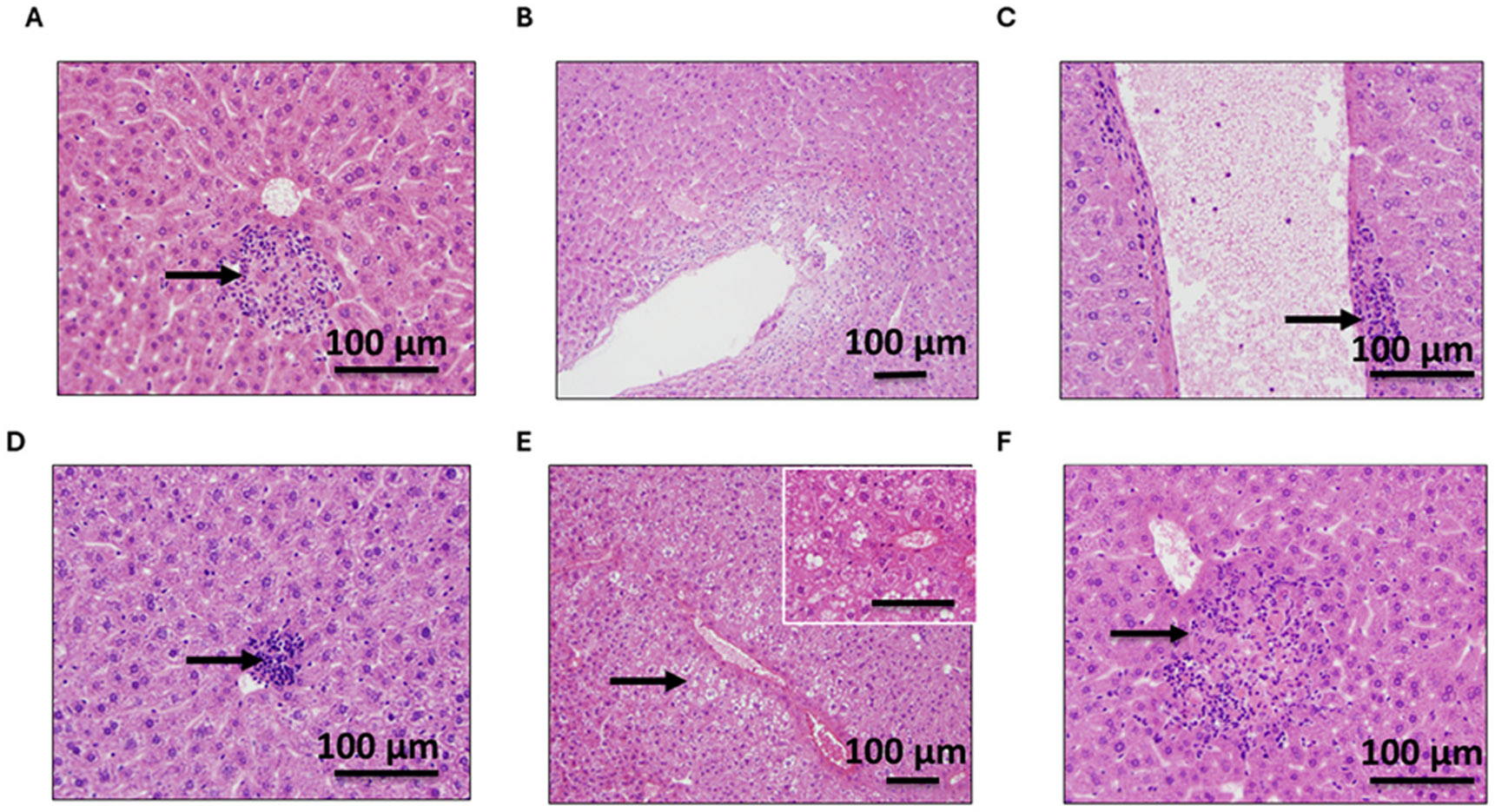
Representative sections for the effect of OC on liver toxicity in nine-month-old 5xFAD mice. (A) Vehicle-treated 5xFAD mouse with focal chronic inflammation (arrow) (20x objective; 200x), (B) 5xFAD mouse receiving 0.5 mg/kg OC showing rare scattered chronic inflammatory cells (10x objective; 100x), (C) 5xFAD mouse receiving 2.5 mg/kg showing focal chronic inflammation adjacent to a vessel (arrow) (20x objective; 200x), (D) 5xFAD mouse receiving 5 mg/kg OC showing focal mild chronic inflammation (arrow) (20x objective; 200x), (E) One 5xFAD mouse receiving 10 mg/kg OC showed focal microvesicular and macrovesicular steatosis (arrow) (10x objective; 100x), and (F) 5xFAD mouse receiving 20 mg/kg OC showing focal acute and chronic inflammation (arrow) with associated rare necrotic hepatocyte (20x objective; 200x). The insert in E is a magnification at 400x to show focal steatosis (E).

Organ toxicity was evaluated in both mouse groups, the prevention and treatment groups. In the prevention group (Fig. 9), no toxicity, ischemia, or necrosis was observed in the small or large intestine and spleen when treated with 0.5–20 mg/kg OC (Supplementary Figures 4-6). Kidney sections were examined for acute tubular injury/necrosis, tubular vacuolization, interstitial fibrosis, and glomerular alterations. None of the mice showed renal toxicity (Fig. 9A and B). One mouse receiving 20 mg/kg OC showed focal subcapsular hemorrhage that was likely procedure-related. Liver sections of both WT and 5xFAD mice with or without OC treatment showed occasional minute areas with mild chronic inflammation. However, the 5xFAD mice receiving 20 mg/kg OC had more frequent foci of mild chronic inflammation and focal microvesicular steatosis (Fig. 9D). Collectively, these results suggest OC is not toxic, except at the high 20 mg/kg dose, where chronic OC could cause hepatic toxicity. In the treatment group, similarly, OC treatment for three months didn’t cause toxicities in the organs examined, the kidney, spleen, small intestine, or large intestine (Supplementary Figures 4-6). Two WT and five 5xFAD mice (across all doses) showed focal mild chronic inflammation in the liver, with one mouse having focal microvesicular and macrovesicular steatosis after receiving 10 mg/kg OC (Fig. 10 A-E). However, hepatic toxicity was more pronounced in the 5xFAD mice receiving 20 mg/kg OC (Fig. 10 F). With 20 mg/kg OC treatment, 5xFAD mice had foci of chronic inflammatory cells with rare focal acute inflammation extending into hepatocytes in one animal (piecemeal necrosis) with an associated apoptotic body.

## 4. Discussion

We previously reported OC reduces brain Aβ plaque load by shifting the amyloid processing pathway to the non-amyloidogenic pathway and by increasing Aβ clearance across the BBB, degradation, and autophagy [16,22,24,25]. Moreover, OC reduces neuroinflammation and BBB dysfunction [16,22,25,35,42]. However, the dose-dependent effect of OC on changes in Aβ, metabolic phenotypes, and organ toxicity remains unknown. Thus, the objectives of this work were to assess the dose-dependent effect of chronic oral OC administered daily for three months in early and advanced AD pathology mouse models for prevention and treatment approaches, respectively, on (1) AD-related metabolic alterations and (2) on brain Aβ levels and organ toxicity. In the current study, the 5xFAD mouse model of AD was used. 5xFAD mice express APP mutations resulting in an early and aggressive deposition of brain Aβ plaque [39,40]. OC treatment was initiated at one-month and six-month-old mice for three months, ending at four-month and nine-month, representing prevention and treatment approaches, respectively. The three-month treatment period was selected based on our previous studies with EVOO and OC as a representation for chronic treatment [22,24,25,42]. The treatment age was selected based on the disease development in the 5xFAD mouse model; treatment at one month, before pathology development, mimics a prevention mode, and treatment at six months of age, after pathology development, mimics a treatment mode. At the end of treatment, mice were used for metabolic phenotype studies, brain Aβ analysis, and tissue histology for toxicity evaluation.

Recently, we reported the effect of three months of treatment with 10 mg/kg oral OC on metabolic changes, Aβ levels, and sleep behavior in 5xFAD mice as a function of age and pathology. At 10 mg/kg dose, OC altered metabolic parameters and reduced Aβ, anxiety-like behavior, and sleep disturbances in the young and older mice with AD pathology, with the effect being greater in the older mice with advanced pathology [35]. In the current study, we evaluated the dose-dependent effect of OC treatment on metabolic and behavioral phenotypes, brain Aβ, and organ toxicity. Our findings demonstrate that three months of treatment with OC altered metabolic and behavioral phenotypes in the 5xFAD mouse, mainly in the 5–20 mg/kg dose range. The effect of OC was more pronounced in the treatment group, i.e., OC treatment after pathology development (nine-month-old mice). However, at 20 mg/kg, OC produced hepatic toxicity, an effect not observed with the lower doses up to mg/kg, suggesting the safety of oral OC at doses less than 20 mg/kg in mice.

Dehydration has been associated with aging and AD due to aging kidneys or lower vasopressin [43,44]. Moreover, due to neuronal dysfunction, water dyshomeostasis occurs early in the disease [45]. In this study, we saw a difference in the dehydration rate (VH_2_O) as a function of pathology when comparing the 5xFAD to WT mice. Young 5xFAD mice demonstrated a reduced VH_2_O compared to the older mice, which could suggest a pathology-dependent alteration. For other metabolic parameters, there is a lack of significant difference between WT mice and 5xFAD mice, which might be attributed to the young age of the mice with low pathology. 5xFAD mice with early AD pathology had energy expenditure and activity that tended to be higher than the WT mice; however, this difference was insignificant. Yet, OC treatment modulated some of these altered metabolic parameters, mainly with OC doses in the range of 5–20 mg/kg. For example, in this dose range, OC reduced VO_2_ and VCO_2_, suggesting reduced ventilation. While the exact mechanism by which OC affects respiratory phenotypes has not been evaluated, it has been shown that OC reduced neuroinflammation linked to intermittent hypoxia in 5xFAD mice [46]. We didn’t assess neuroinflammation in this study; however, our previous work showed OC to reduce neuroinflammation after three months of treatment with OC and OC-rich olive oil [16,22,25,26].

Within the treatment group, the effect of pathology on metabolic phenotypes was more evident, as published previously [35]. The low doses of OC in the studied range (0.5–2.5 mg/kg) were insufficient to alter 5xFAD mice’s metabolic parameters. However, the higher doses of OC restored the metabolic parameters to levels similar to those of the WT mice. 5xFAD mice had a higher dehydration rate and energy expenditure than WT mice, which could be related to pathology development. The exact mechanism by which 5xFAD mice develop dehydration requires further studies. However, with advanced pathology, we speculate that the increased dehydration (VH_2_O) could be attributed to a diminished response to acetylcholine with reduced vasopressin levels as the pathology progresses. Moreover, compared to WT mice, 9-month-old 5xFAD mice with advanced AD demonstrated hyperventilation, higher RER, and abnormal respiratory phenotypes, which could be attributed to altered ventilatory chemoresponses [47]. Furthermore, advanced pathology 5xFAD mice had faster and longer distances traveled, which suggests depression and anxiety-like behavior [48,49]. Indeed, to confirm these findings, the effect of OC on memory, depression, and anxiety should be performed. While we previously reported the positive effect of 3-month treatment of 5xFAD and TgSwDI mouse models of AD with OC-rich EVOO and EVOO-derived phenolics [22,25,42], studies focus on evaluating the dose-dependent effect of OC on depression and anxiety as well as memory are essential.

Sleep disturbances are associated with normal aging [50]. However, with AD pathology, sleep disturbances are more pronounced and could contribute to pathology exacerbations[38]. Reduction in night sleep duration and daytime sleepiness are reported to affect cognitive abilities and cause dementia [51,52]. Besides, sleep disturbances develop as the disease progresses [53]. Initially, it starts as a disturbance in the circadian rhythm, followed by a decrease in night sleep time and inability to fall back to sleep, and ends with increased daytime sleepiness and napping [54]. 5xFAD sleep disturbances could be related to respiratory phenotype changes, as sleep disturbances in 5xFAD mice older than 8 months are linked to apnea and ventilatory responses during sleep [55]. Sleep disturbances in 5xFAD mice are inconsistently reported in the literature, where it has been shown to start at the age of 4–4.5 months old mice in some studies [56,57], including a reduction in night-time sleep by 12 % compared to control mice [56]. Discrepancies in sleep data can be attributed to gender differences, where female mice had more sleep disturbances than males, or attributed to different study designs [56,57]. However, in our study, we did not see any difference in daytime and nighttime sleep at 4 months of age, which is consistent with our previous work comparing WT and 5xFAD mice [35]. The current study showed sleep disturbances between WT and 5xFAD mice with advanced pathology. Low doses of 0.5–2.5 mg/kg OC were insufficient to rectify total sleep hours. However, higher doses of 5–20 mg/kg increased the sleep hours, reaching the WT mice total hours.

We have shown the beneficial effects of oleocanthal in lowering brain Aβ load previously [16,25,35,42]. In the current work, we performed a dose-dependent study to evaluate the effect of OC in lowering Aβ levels. OC treatment reduced soluble Aβ42 (while insignificant) and Aβ_40_ starting at doses as low as 0.5 mg/kg. 5xFAD mice express mutations leading to the overproduction of Aβ [58], and based on our published findings, Aβ reduction could be related to increased clearance [16]. We have shown previously that OC improves the clearance of Aβ by rectifying its clearance across the blood-brain barrier and increasing the expression of Aβ enzymatic degradation enzymes and autophagy [16,22,24,42]. However, based on OC effect on metabolic parameters and Aβ brain levels, no link could be determined between the 2 measurements; OC seemed effective in reducing Aβ brain levels at low doses, including the 0.5 mg/kg dose in the prevention and treatment groups, an effect that was not associated with metabolic parameters alterations which required much higher doses for the effect to be observed.

Previously, researchers have shown that hypothalamus Aβ levels in 6-month-old 5xFAD mice are associated with decreased body weight, food intake, and energy expenditure [59]. We found no differences in body weight in mice with early pathology; this aligns with the lack of plaques in mouse brains. However, 5xFAD mice demonstrated a significant weight loss with advanced pathology compared to age-matched WT mice, which was rectified by OC treatment except with the 20 mg/kg OC, an effect that could be attributed to the observed hepatic toxicity in 5xFAD mice receiving 20 mg/kg OC.

Compared to the cognitively normal individuals of the same age, AD patients have lower body weight due to appetite and metabolic state changes [60,61]. In addition, a study by Jang and colleagues reported that overweight AD patients had a lower mortality risk compared to normal weight patients, with the underweight AD patients having the highest mortality risk [62]. Moreover, in 5xFAD mice, a positive correlation between body weight and performance in motor behaviour tests has been reported at 12–16 months of age [63]. Similarly, in our study, we observed a lower body weight in 9-month-old 5xFAD mice compared to WT mice, which was increased by OC treatment, suggesting the protective effects of OC in AD. While additional studies are required to explain the increased body weight in the treatment mode group, one could speculate that the increased body weight could be due to increased food consumption due to reduced pathology-associated memory dysfunction. According to Supplementary Figure 3, OC didn’t alter food intake; however, studies have shown that an acclimation period of up to 5 days could be necessary to monitor changes in feeding and drinking [64], and in our study, we used a 12-hour acclimation period in singly housed cages, which may not be enough acclimation time that collectively affected the accurate measurement of food and water intake, and possibly the body weight.

We evaluated the dose-dependent toxicities of OC. Our findings demonstrate up to 10 mg/kg, OC is relatively safe in mice, and OC only produced hepatic toxicity at the highest dose of 20 mg/kg. OC showed no gastrointestinal toxicities in the small and large intestines or the spleen. A few mice in the prevention group and treatment group had minute hemorrhages. However, this observation was inconsistent, as it was also observed in WT mice and might be a procedure-related issue. Additionally, the effects could be related to the chronic oral gavage performed for 3 months, which might have stressed the mice and caused such observations. Furthermore, it has been reported that external stress in rodents can influence heart rate and blood pressure [65] and may lead to early cognitive deficits and increased Aβ pathology [66] potentially confounding the experimental results. To reduce these effects, both control groups—the WT and 5xFAD mice—received the vehicle by oral gavage for 3 months, matching OC dosing, to ensure similar conditions.

However, OC demonstrated hepatic toxicities at a 20 mg/kg dose and was more pronounced in the treatment group, i.e., with the older mice with advanced AD pathology. Findings from a previous report suggested the single acute oral dose of 250 mg/kg OC as the upper possible dose [23]. Here, we report that the chronic administration of oral 20 mg/kg OC demonstrates hepatotoxicity when administered daily for 3 months. While OC possesses anti-inflammatory effects at low doses [25], OC chronic dosing causes mild acute and chronic inflammation with focal hepatocyte necrosis at 20 mg/kg, which suggests limiting the chronic dosing of OC up to 10 mg/kg orally in mice.

It has been documented in the literature that male and female WT mice have metabolic differences [67], and that female 5xFAD mice have higher levels of brain Aβ, more neuroinflammation, different sleep cycles, reduced glycolysis in the auditory cortex, and more deficits in object recognition and social exploration than male 5xFAD mice [48,68]. Therefore, our results may not be generalized to female 5xFAD mice, as gender-dependent phenotypic changes are present. Thus, studies that include male and female mice to evaluate OC are necessary. Another limitation is the absence of dose-dependent OC in WT mice, as OC is currently available as a dietary supplement for the general population. Thus, additional studies are required to assess the toxicity of OC in WT mice. Finally, further studies are necessary to clarify the underlying mechanism(s) by which OC affects metabolic parameters, energy expenditure, and sleep patterns.

In conclusion, we observed that OC caused metabolic and behavioral phenotypic changes and reduced brain Aβ levels in the examined dose range to a variable extent. Moreover, our findings reveal hepatic toxicity following three months of daily treatment with 20 mg/kg. Toxicities were more pronounced in the older mice with advanced pathology, suggesting OC toxicity might be pathology-related. However, the effect of OC, duration, and age should not be excluded as contributing factors to the toxicity; indeed, additional investigations are necessary to clarify each factor’s contribution. Finally, our findings indicate that the metabolic and behavioral parameters assessed in this study could be modulated by OC treatment. However, the dosage must be tuned to exert beneficial effects without inducing toxicity. Therefore, based on our findings, we suggest the 5 and 10 mg/kg oral OC doses to rectify metabolic and phenotypic changes associated with AD in the 5xFAD mouse model.

## Supplementary Material

Supp data

## Figures and Tables

**Table 1:** A summary of the assessed metabolic parameters with their abbreviations.

Parameter	units	Definitions
Body weight	gram (g)	Mouse mean body mass
Food intake	gram (g)	Mean mass of food consumed by the mouse
Water intake	gram (g)	Mean mass of water consumed by the mouse
Sleeping time	hours (h)	Cumulative sleep duration. An animal was considered sleeping when it exhibited "quiet" behavior exceeding 40 s. "Quiet" time was defined as the duration during which the animal did not engage in eating, drinking, grooming, or locomotion.
VH_2_O	milliliters per minute (mL/min)	Water vapor loss
VO_2_	milliliters per minute (mL/min)	Mean rate of Oxygen consumption
VCO_2_	milliliters per minute (mL/min)	Mean rate of Carbon dioxide consumption
RER	Unitless	The respiratory quotient is the ratio of VCO_2_ to VO_2_. RER was determined by measuring gas exchange within the metabolic cages to identify the primary energy substrate. Specifically, RER is the ratio of CO2 produced to the volume of O2 consumed (RER = VCO_2_/VO_2_).
EE	kilocalories per hour (kcal/hr).	Energy expenditure (EE) was calculated in kilocalories (kcal) utilizing the Weir equation: 60*(0.003941VO_2_ (n) + 0.001106VCO_2_ (n)), where VO_2_ and VCO_2_ are measured in mL/min.
Cumulative distance	meters	The cumulative distance traveled

## Data Availability

Data will be made available on request.

## Appendix A. Supporting information

Supplementary data associated with this article can be found in the online version at doi:10.1016/j.prenap.2025.100357.
