# Supplementary material for: Dose-dependent evaluation of chronic oleocanthal on metabolic phenotypes and organ toxicity in 5xFAD mice
Source: Pharmacol Res Nat Prod. Author manuscript; Available in PMC 2026 Jan 27. (PMC12833832; doi:10.1016/j.prenap.2025.100357)
Supplement: Supp data [file NIHMS2136869-supplement-Supp_data.pdf]

Dose-dependent effect of chronic oleocanthal on metabolic phenotypes and organ toxicity in 5xFAD mice

Euitaek Yang[1†], Nour F. Al-Ghraiybah[1†], Amer E. Alkhalifa[1], Lauren N. Woodie,[2] Samuel P. Swinford,[1] Judy King,[3] Michael W. Greene[2], Amal Kaddoumi[1]*

[1] Department of Drug Discovery and Development, Harrison College of Pharmacy, Auburn University; Auburn, AL, 36849, USA.

[2] Department of Nutritional Sciences, College of Human Sciences, Auburn University; Auburn, AL, 36849, USA.

[3] Department of Basic Sciences, DeBusk College of Osteopathic Medicine, Lincoln Memorial University, Knoxville, TN, 37932, USA

[4] Department of Pharmacology and Toxicology, Medical College of Georgia, Augusta University, Augusta, GA, 30912, USA

† These authors contributed equally to this work

* Corresponding Author

Current contact: Amal Kaddoumi (akaddoumi@augusta.edu), Department of Pharmacology and Toxicology, Medical College of Georgia, Augusta University, 1201 Goss Lane, Augusta, GA 30912. Phone +1-706-723-4439

Supplementary Figure 1. The dose-dependent effect of OC on body weight in (A) four-month-old, and (B) 9-month-old mice. Data are presented as mean ± SEM for n=10 per group with *p<0.05 compared to vehicle-treated 5xFAD mice, and #p<0.05 and ##p<0.01 compared to WT mice.

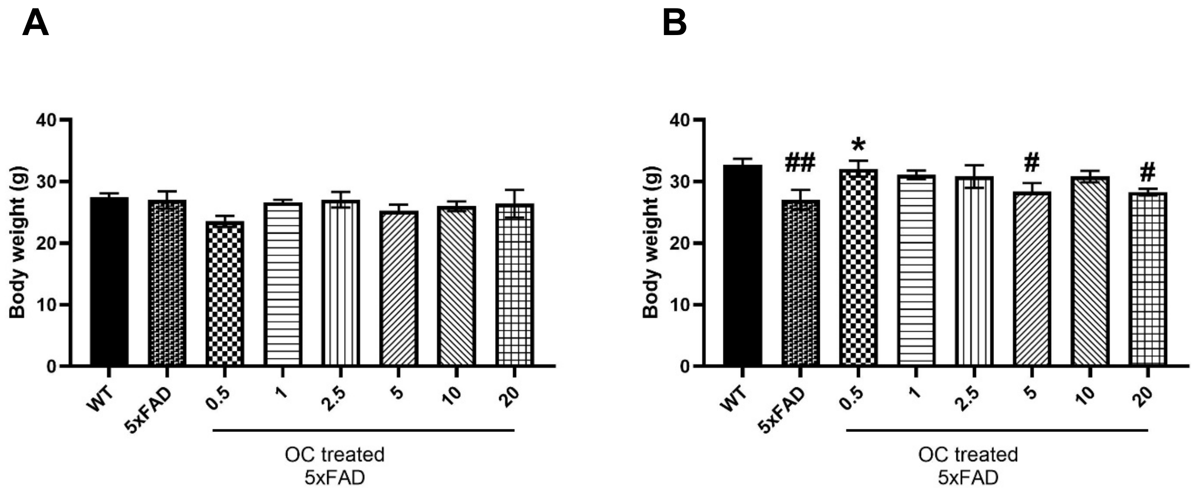

Supplementary Figure 2. The dose-dependent effect of OC on food and water intake in four-month-old mice. (A) Food intake at nighttime (g), (B) water intake at nighttime (g), (C) food intake at daytime (g), and (D) water intake at daytime (g). Data are presented as mean ± SEM for n=10 per group with **p<0.01 compared to vehicle-treated 5xFAD mice.

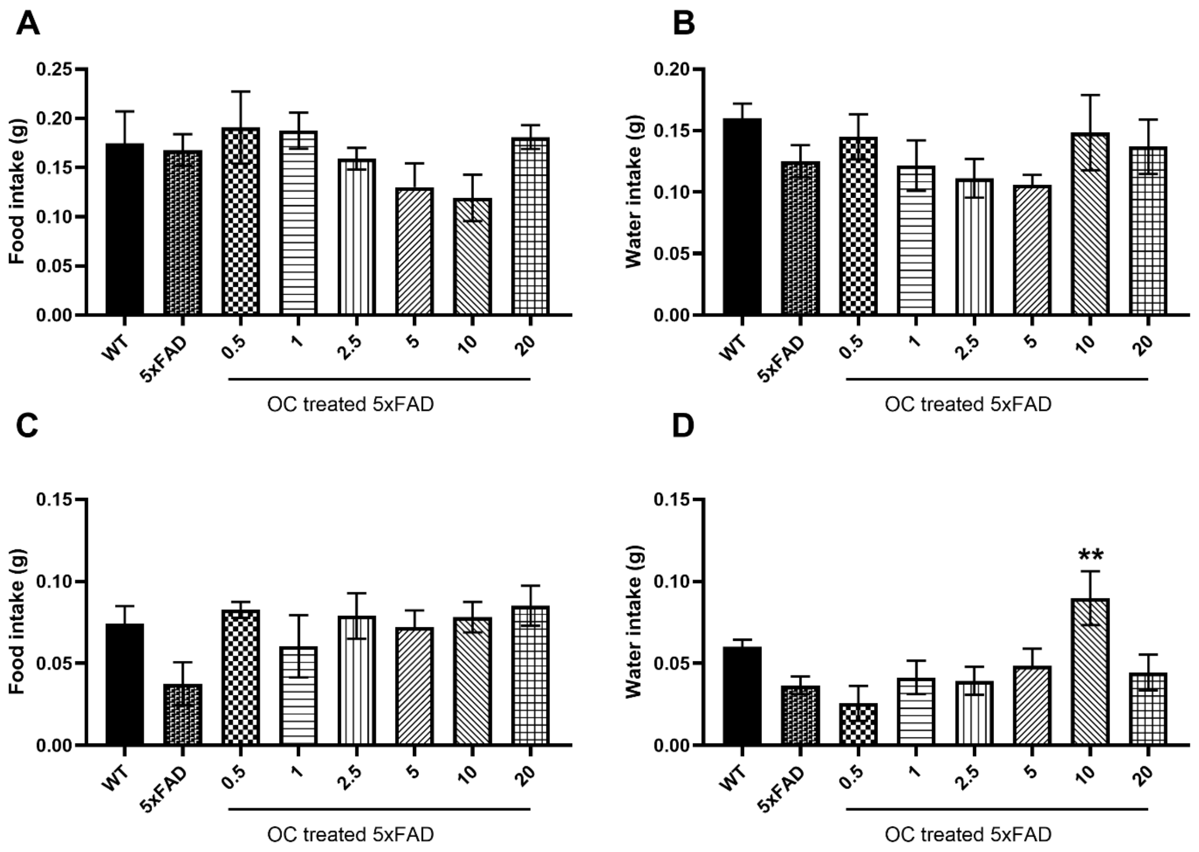

Supplementary Figure 3. The dose-dependent effect of OC on food and water intake in nine-month-old mice. (A) Food intake at nighttime (g), (B) water intake at nighttime (g), (C) food intake at daytime (g), and (D) water intake at daytime (g). Data are presented as mean ± SEM for n=10 per group with #p<0.05 compared to vehicle-treated WT mice.

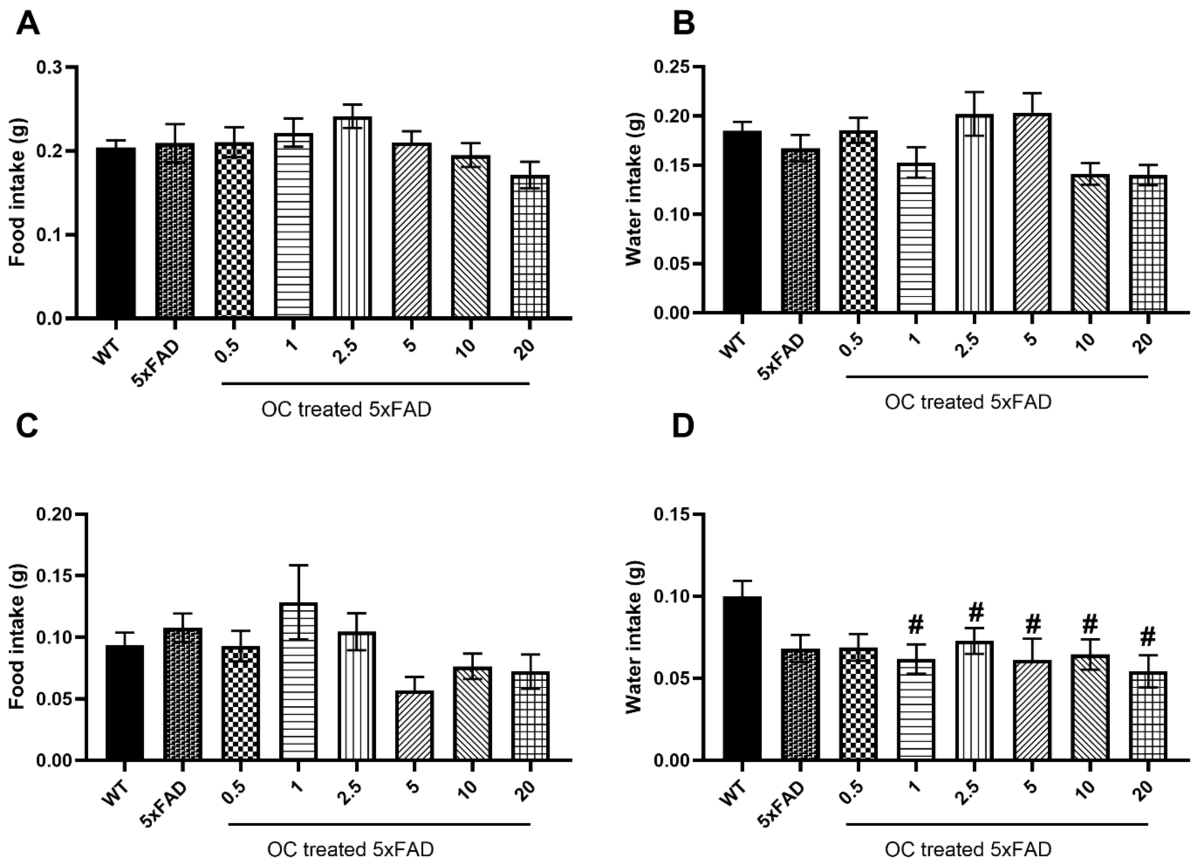

Supplementary Figure 4. Representative images of OC effect on the spleen of (A) four-month-old mice, and (B) nine-month-old mice.

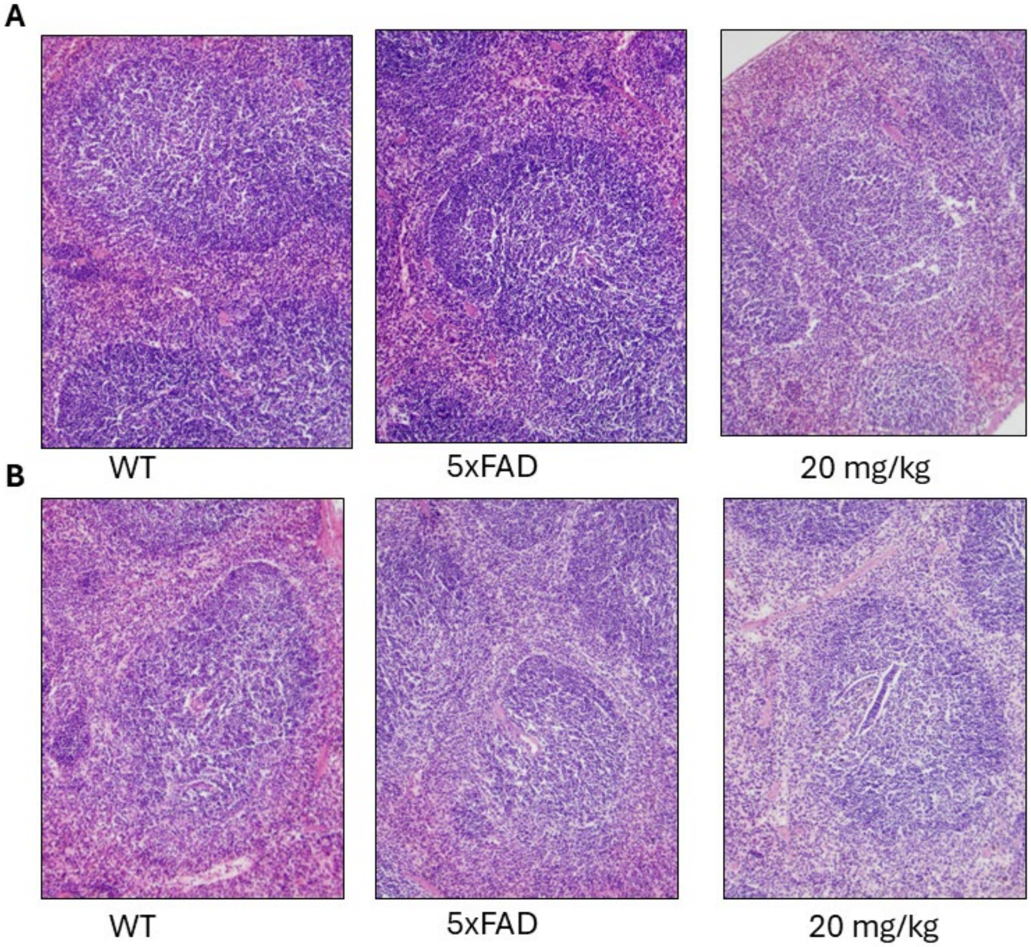

Supplementary Figure 5. Representative images of OC effect on the small intestine of (A) four-month-old mice, and (B) nine-month-old mice.

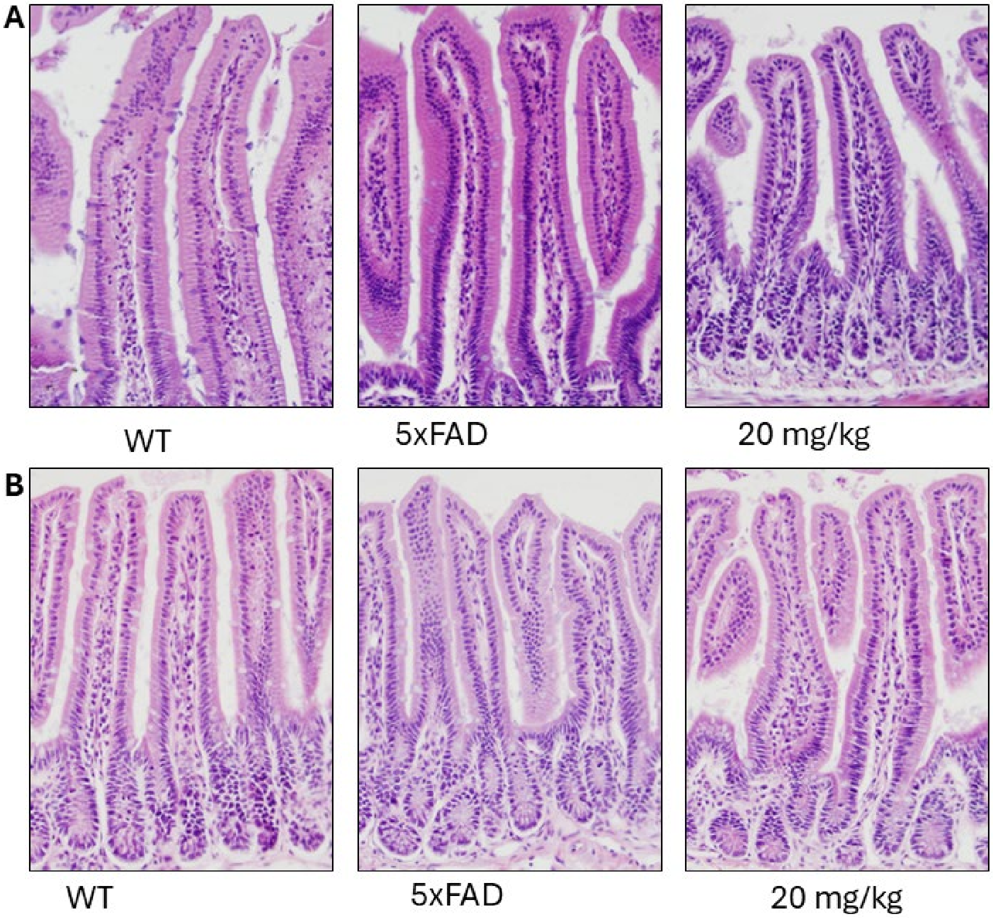

Supplementary Figure 6. Representative images of OC effect on the large intestine of (A) four-month-old mice, and (B) nine-month-old mice.

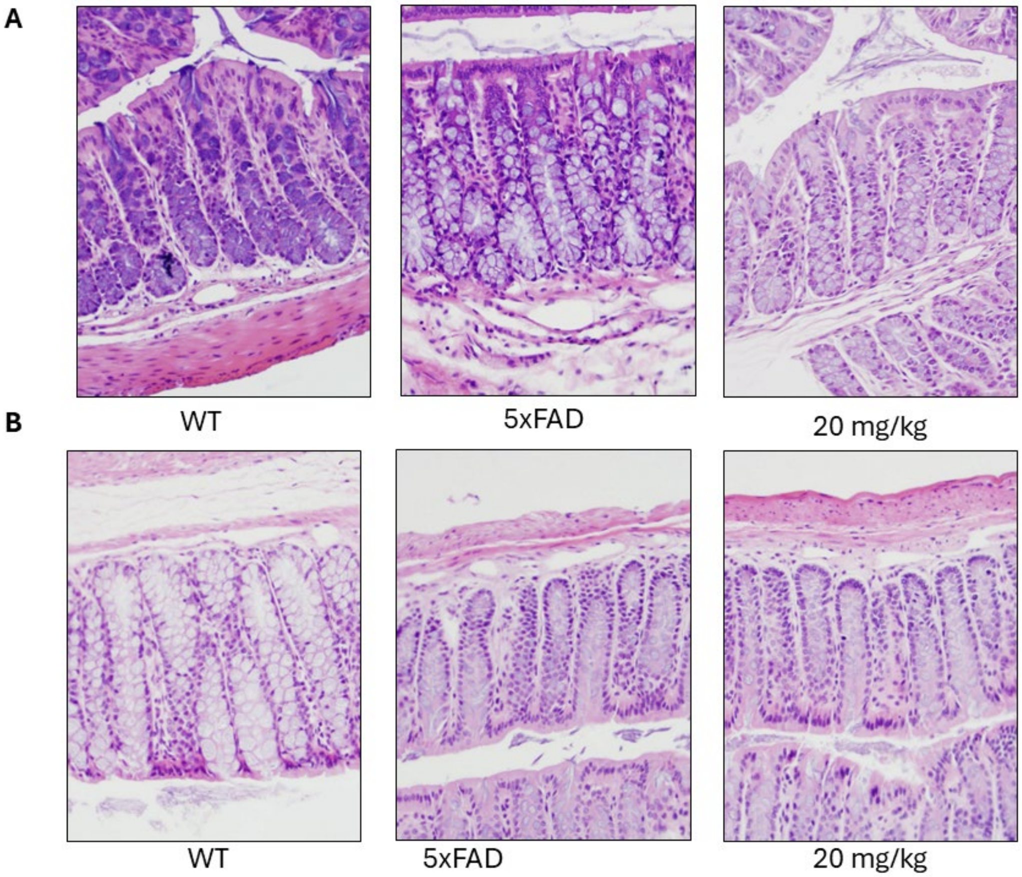

Supplementary Table 1. Statistical parameters following the one-way ANOVA analysis of the 8 groups for each measured variable.

4-month-old mice: Nighttime

		df	F	P-value
Ave_VO2	Between Groups	7	5.435	.000
	Within Groups	42		
	Total	49		
Ave_VCO2	Between Groups	7	5.902	.000
	Within Groups	42		
	Total	49		
Ave_VH2O	Between Groups	7	4.076	.002
	Within Groups	42		
	Total	49		
Ave_EE	Between Groups	7	5.545	.000
	Within Groups	42		
	Total	49		
Ave_RER	Between Groups	7	5.781	.000
	Within Groups	42		
	Total	49		
Ave_Cum. Distance	Between Groups	7	3.996	.002
	Within Groups	42		
	Total	49		

4-month-old mice: Daytime

		df	F	P-value
Ave_VO2	Between Groups	7	1.497	.195
	Within Groups	42		
	Total	49		
Ave_VCO2	Between Groups	7	1.125	.366
	Within Groups	42		
	Total	49		
Ave_VH2O	Between Groups	7	4.355	.001
	Within Groups	42		
	Total	49		
Ave_EE	Between Groups	7	1.404	.229
	Within Groups	42		
	Total	49		
Ave_RER	Between Groups	7	2.094	.065
	Within Groups	42		
	Total	49		
Ave_Cum. Distance	Between Groups	7	3.952	.002
	Within Groups	42		
	Total	49		

9-month-old mice: Nighttime

		df	F	P-value
Ave_VO2	Between Groups	7	26.632	.000
	Within Groups	71		
	Total	78		
Ave_VCO2	Between Groups	7	36.591	.000
	Within Groups	71		
	Total	78		
Ave_VH2O	Between Groups	7	4.694	.000
	Within Groups	71		
	Total	78		
Ave_EE	Between Groups	7	29.047	.000
	Within Groups	71		
	Total	78		
Ave_RER	Between Groups	7	10.713	.000
	Within Groups	71		
	Total	78		
Ave_Cum. Distance	Between Groups	7	9.475	.000
	Within Groups	71		
	Total	78		

9-month-old mice: Daytime

		df	F	P-value
Ave_VO2	Between Groups	7	28.925	.000
	Within Groups	71		
	Total	78		
Ave_VCO2	Between Groups	7	37.265	.000
	Within Groups	71		
	Total	78		
Ave_VH2O	Between Groups	7	5.831	.000
	Within Groups	71		
	Total	78		
Ave_EE	Between Groups	7	31.282	.000
	Within Groups	71		
	Total	78		
Ave_RER	Between Groups	7	10.079	.000
	Within Groups	71		
	Total	78		
Ave_Cum. Distance	Between Groups	7	6.886	.000
	Within Groups	71		
	Total	78		
